# Supplementary material for: Patterns of Intron Gain and Loss in Fungi
Source: PLoS Biol. 2004 Nov 30;2(12):e422. doi: 10.1371/journal.pbio.0020422 (PMC532390; doi:10.1371/journal.pbio.0020422)
Supplement: Table S1 — Also available at http://genes.mit.edu/NielsenEtAl/. (4.3 MB ZIP). [file pbio.0020422.st001.zip › NielsenEtAl/html/357.html]

AN5875.1.NCU07041.1.MG06049.1.FG06203.1


```
 CLUSTAL W (1.82) Multiple Sequence Alignments - Introns Inserted


Sequence 1: AN5875.1	348 aa
Sequence 2: MG06049.1	320 aa
Sequence 3: NCU07041.1	320 aa
Sequence 4: FG06203.1	426 aa
Alignment Length: 454 aa
Number Identitical Residues: 202 aa
Alignment Score (without introns) 9253


MG06049.1 	------------------------------------------------------------
NCU07041.1	------------------------------------------------------------
FG06203.1 	MGYDVPPGCPTDQTFFSSSLNCTQLRKKNLRVASFTNFSFSTAVLRNNLHYAPLTQENLG
AN5875.1  	------------------------------------------------------------
          	                                                            

MG06049.1 	----------------------------------------------MPSTHNVDKPWDTE
NCU07041.1	----------------------------------------------MPSTHKKEKPWDTD
FG06203.1 	VSSLTLSRLVFLQAVLRNNLDSAQLIQDKPGLALDTQPASEYTSTTMPSTHNVAKPWDTE
AN5875.1  	----------------------------------------------MPSTNNKDKPWDTD
          	                                              ****::  *****:

MG06049.1 	DIDKWK~VDPFTPSDNAGGTFAEESSFMTLFPKYREVYLRQSWPLVTKALEKYGIATTLD
NCU07041.1	DIDKWK~VDPFT-KDESSGPFLEESSFMTLFPKYRERYLRDSWPLITKALDKHGITAVLD
FG06203.1 	DIDKWR~IEPFLPKDNVGGPFTEESSFATLFPKYRESYLKEVWPLITRALEKLGIACSLD
AN5875.1  	DIDKWK0IEEFKPEDNAGGSFAEESSFATLFPKYREVYLKEAWPVVTRALEKHGIACTLD
          	*****: :: * ..*: .*.* ***** ******** **:: **::*:**:* **:  **

MG06049.1 	LVEGSMTVKTTRK~TFDPAAILNARDLIKLLARSVPAPQ~AIKILEDGVACDIIKIRNLV
NCU07041.1	LVEGSMTVKTTRK0TYDPAAILNARDLIKLLARSVPAPQ~AIKILEDGMACDIIKIRSMV
FG06203.1 	LIEGSMVVKTSLR~THDPSSILKARDLIKLLARSVPAPQ~AIKILEDGIACDIIKIRNQV
AN5875.1  	LVEGSMTVKTTRK~TYDPAAILKARDLIKLLSRSVPVQQ0ALKILEDGVACDIIKIRNQV
          	*:****.***: : *.**::**:********:****. * *:******:********. *

MG06049.1 	RNKERFIKRRQRILGPNGSTLK~ALELLTSTYILVHGNTVSAMGPYKGLKEVRRVVEDCM
NCU07041.1	RNKERFVKRRQRILGQNGTTLK~ALELLTQTYILVHGNTVSVMGPFKGLKEVRRVVEDTM
FG06203.1 	ASKERFVKRRQRLLGPNGTTLK0ALELLTKTYILVHGNTVSVMGPYKGLKEIRRVVEDCM
AN5875.1  	RNKERFVKRRQRILGPQGSTLK~ALELLTGTYILVQGNTVSAMGPYKGLKEVRRIVDDCM
          	 .****:*****:** :*:*** ****** *****:*****.***:*****:**:*:* *

MG06049.1 	NNVHPIYLIKELM~VKRELAKDPALANESWDRFLPNFKKRSLSHRRVPHKVNDKSKKTYT
NCU07041.1	NNVHPIYMIKELM2IKRELAKDPALAHEDWSRYLPNFKKRTLSKRRVPHVVNDKTKKNYT
FG06203.1 	ANIHPIYHIKEAM~IKQELAKDPELANESWDRFLPNFKRKTLSQRRVPHKVNDKAKKVYT
AN5875.1  	ANIHPIYHIKELM~IKRELAKDPTLAHESWDRFLPNFKKRTLSKRRTPFKVTDKSKKVYT
          	 *:**** *** * :*:****** **:*.*.*:*****:::**:**.*. *.**:** **

MG06049.1 	AFPPAPEKSKVDLQIESGEYFLGKEAKKRAAETERQEQAKVKKEERKREREKDFVPPEE-
NCU07041.1	PFPPAPEKSKVDLQIESGEYFLGKEAKQRVAEQERAEKAKQKKEEKKREREKEYVPPEE-
FG06203.1 	PFPPAPEKSKVDKQIESGEYFLGKVGKERAAQEERREKQNKRKEEKAKEREADFIAPEE-
AN5875.1  	PFPPAPEKSKVDLQIESGEYFLSKEAKERAQKEEVMERQRIKREEKMKEREKAFVAPEEL
          	.*********** *********.* .*:*. : *  *: . ::**: :***  ::.*** 

MG06049.1 	--------------------PG~TQTKKRKTKA-------
NCU07041.1	-------------------LTD~KKAKKRKTSD-------
FG06203.1 	--------------------SR~PKKKRKKTEE-------
AN5875.1  	EETEKAKKEKKEKKKKRKRDSE0KEKKKKKSKSKAESDDE
          	..:..:.......... .  .   : *::*:. .:.:...
```
